# Supplementary material for: Silicon-Selenium Interplay Imparts Cadmium Resistance in Wheat through an Up-Regulating Antioxidant System
Source: Int J Mol Sci. 2023 Dec 27;25(1):387. doi: 10.3390/ijms25010387 (PMC10778846; doi:10.3390/ijms25010387)
Supplement: Supplementary file 1 [file ijms-25-00387-s001.zip › ijms-2734427-supplementary.pdf]

Supplementary data

Table S1: Composition of nutrient solution used in the experimental work.

Salts	Molecular weight	Concentration in nutrient solution
$Ca(NO_3)_2 \cdot 4\ H_2O$	236.15	2 mM
K_2SO_4	174.27	1 mM
$MgSO_4 \cdot 7\ H_2O$	246.48	0.5 mM
$CaCl_2 \cdot 2\ H_2O$	147.02	2 mM
KH_2PO_4	136.09	0.2 mM
Fe-EDTA	367.05	0.2 mM
H_3BO_3	61.83	10 µM
$MnSO_4 \cdot H_2O$	169.02	2 µM
$ZnSO_4 \cdot 7H_2O$	287.55	0.5 µM
$CuSO_4 \cdot 5H_2O$	249.68	0.3 µM
$(NH_4)Mo_7O_{24}$	1235.86	0.01 µM

Table S2: Effect of Se and Si on elemental composition of macro and micro elements for uptake in root and shoot of wheat under Cd stress. Data presented in the mean of 4 replicates± standard error. A (*) represents statistical difference at p>0.05.

**Elemental composition▶**	**Cd** (mg kg^{-1})	**Se** (ug kg^{-1})	**Si** (ug kg^{-1})	**Zn** (mg kg^{-1})	**Cu** (mg kg^{-1})	**Mn** (mg kg^{-1})	**Ca** (g kg^{-1})	**Mg** (g kg^{-1})	**P** (g kg^{-1})	**S** (g kg^{-1})	**Fe** (g kg^{-1})	**Na** (g kg^{-1})
**Treatments▼**						**Root**						
**Control**	0.12±0.0	15.93±1.0	0.9±0.2	*4.01±0.4	3.68±0.3	5.02±1.0	48.91±11.4	*0.53±0.1	0.44±0.1	*0.78±0.1	2376±382	*1050±66
**Se**	0.09±0.0	*72.06±8.3	0.8±0.1	3.41±0.2	3.09±0.4	6.31±1.6	49.38±27.1	0.39±0.1	0.50±0.0	0.76±0.1	*3761±570	1002±126
**Se+Si**	0.71±0.4	55.95±2.5	1.8±0.1	2.90±0.1	4.71±0.9	5.17±0.5	18.046.3	0.43±0.1	0.47±0.1	0.67±0.1	2954±257	861±194
**Cd**	*840.8±55.3	23.17±0.6	0.6±0.1	2.16±0.2	3.42±0.5	5.71±1.0	37.42±7.5	0.48±0.1	0.39±0.1	0.63±0.0	3494±514	978±42.2
**Cd+Se**	362.1±47.0	34.87±4.2	0.9±0.1	1.46±0.1	2.21±0.3	6.93±1.3	32.11±4.1	0.35±0.1	0.40±0.0	0.46±0.0	2512±241	389±55
**Cd+Si**	472.4±17.3	22.10±1.5	2.1±0.3	2.15±0.1	*5.18±0.8	*8.31±1.3	34.30±7.2	0.44±0.1	0.45±0.0	0.57±0.0	3246±339	710±101
**Cd+Se+Si**	325.4±31.3	49.83±5.4	*2.3±0.1	2.00±0.1	3.40±0.7	6.72±1.0	25.76±3.6	0.40±0.1	*0.60±0.1	0.46±0.1	2858±404	641±52
						**Shoot**						
**Control**	0.02±0.0	7.65±1.1	0.5±0.1	3.20±0.1	1.05±0.1	9.37±1.1	0.96±0.1	0.26±0.1	0.40±0.0	0.49±0.0	15±1.7	22±2.3
**Se**	0.01±0.0	*50.37±10.1	0.7±0.1	*3.52±0.5	1.21±0.2	11.86±1.8	1.24±0.2	0.28±0.1	0.47±0.1	0.56±0.1	24±4.0	20±2.8
**Se+Si**	0.07±0.0	29.99±0.9	1.2±0.2	3.48±0.4	0.70±0.1	12.61±1.2	1.22±0.1	0.25±0.1	0.47±0.1	0.58±0.1	18±0.9	17±1.8
**Cd**	*65.4±4.6	9.58±1.9	0.5±0.1	2.94±0.2	1.28±0.1	8.12±0.5	1.18±0.1	0.32±0.1	0.30±0.1	0.56±0.1	*22±2.0	22±1.3
**Cd+Se**	47.0±3.3	29.47±2.5	0.5±0.1	3.47±0.2	0.90±0.1	12.05±1.5	1.26±0.1	0.29±0.1	0.45±0.1	*0.65±0.1	*22±0.8	18±1.3
**Cd+Si**	41.2±6.3	8.28±1.2	*1.6±0.2	3.48±0.3	1.00±0.2	*12.34±1.7	1.30±0.2	0.31±0.1	0.46±0.1	0.61±0.1	19±2.5	21±33
**Cd+Se+Si**	30.9±2.6	25.42±3.8	1.4±0.1	2.47±0.4	0.70±0.1	8.60±1.1	0.86±0.2	0.19±0.1	0.41±0.1	0.42±0.1	13±2.5	14±1.4

Table S3: Input file of ionic composition in nutrients solution for possible chemical interaction study in Visual MINTEQ 3.1 .

**0.2 Hoagland's Solution**																				
**Stock Solution**	mM	Ca	NO^{3-}	K	NH_4	PO_4^{-3}	Mg	SO_4	Cl^{-1}	H_3BO_3	Mn	Cu	Zn	MoO_4^{-2}	Fe	EDTA	Na	SeO_3^{-2}	SiO_3^{-2}	Cd^{+2}
$Ca(NO_3)_2 \cdot 4H_20$	2	2	4																	
K_2SO_4	1			1				0.5												
$Mg(SO_4) \cdot 7H_20$	0.5						0.5	0.5												
$CaCl_2.2H_2O$	2	2							4											
KH_2PO_4	0.2			0.2		0.2														
Fe-EDTA	0.2							0.1							0.2	0.2				
H_3BO_3	0.01									0.01										
$MnSO_4 \cdot H_20$	0.0045							0.002			0.002									
$CuSO_4 \cdot 5H_20$	0.0003							0.0003				0.0003								
$ZnSO_4 \cdot 7H_20$	0.0015							0.0005					0.0005							
$(NH_4)_6Mo_7O_{24} \cdot 4H_20$	0.0001				0.00001									0.00001						
Na_2SeO_3	0.0015																0.0032	0.0015		
Na_2SiO_3	0.002																0.004		0.002	
$CdCl_2$	0.01								0.02											0.01
	**Pura**	**4**	**4**	**1.2**	**0.00001**	**0.2**	**0.5**	**1.1028**	**4.02**	**0.01**	**0.002**	**0.0003**	**0.0005**	**0.00001**	**0.2**	**0.2**	**0.0072**	**0.0015**	**0.002**	**0.01**

Table S4: Interaction of Si-Cd in nutrient solution.

Treatments	% of total concentration	Species name
Si	97.21	H_4SiO_4
	0.023	$H_4SiO_4SO_4^{-2}$
	2.766	$H_3SiO_4^{-}$
Cd	1.692	Cd^{+2}
	0.015	$CdOH^{+}$
	0.394	$CdCl^{+}$
	0.121	**$CdSO_4$ (aq)**
	0.013	$CdNO_3^{+}$
	0.305	**$CdHPO_4$ (aq)**
	97.451	$CdEDTA^{-2}$
Cd+Si	97.625	H_4SiO_4
	0.023	$H_4SiO_4SO_4^{-2}$
	2.352	$H_3SiO_4^{-}$
	1.853	Cd^{+2}
	0.015	$CdOH^{+}$
	0.429	$CdCl^{+}$
	0.132	**$CdSO_4$ (aq)**
	0.014	$CdNO_3^{+}$
	0.336	**$CdHPO_4$ (aq)**
	97.212	$CdEDTA^{-2}$

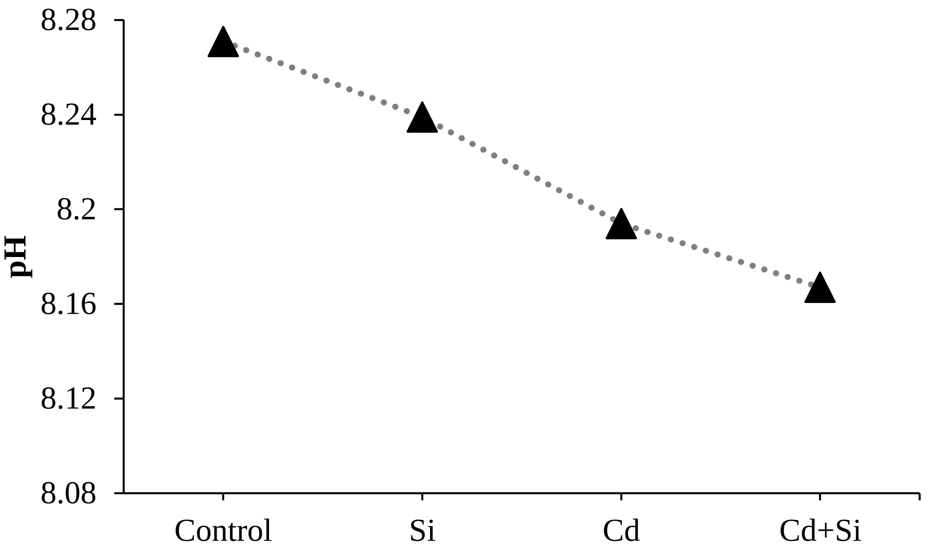

Figure S1: Effect of Si-Cd addition on pH change in nutrient solution inferred by visual MINTEQ 3.1
